# SUMOylation Inhibitor TAK-981 Alleviates Hallmark Features of Preeclampsia Related to High Glucocorticoid Exposure by Inhibiting Placental Oxidative Stress in Rats

**DOI:** 10.3390/antiox15040478

**Published:** 2026-04-11

**Authors:** Shu Xiao, Youyi Zhang, Zhengshan Tang, Caiyun Chen, Dewei Guo, Jing Long, Xin Ni

**Affiliations:** 1 Department of Gynecology and Obstetrics, Xiangya Hospital Central South University, Changsha 410000, China; 2 National Clinical Research Center for Geriatric Disorders, Xiangya Hospital Central South University, Changsha 410000, China; 3 National International Joint Research Center for Medical Metabolomics, Xiangya Hospital Central South University, Changsha 410000, China; 4 Department of Gynecology and Obstetrics, General Hospital of Western Theater Command of PLA, Chengdu 610083, China

**Keywords:** preeclampsia, SUMOylation, placenta, trophoblast, oxidative stress

## Abstract

SUMOylation may be involved in preeclampsia (PE) progression. We aimed to investigate the roles of SUMOylation in PE and its underlying mechanism using animal and cell models. A rat PE model was established by dexamethasone (DEX) treatment from pregnancy day 7.5–17.5. HTR8 and BeWo trophoblasts were used as cell models. Placental RNA-seq analysis coupled with Western blotting showed upregulated SUMOylation in placentas of DEX-treated rats. SUMOylation inhibitor TAK-981 treatment robustly alleviated PE-like features including reduced blood pressure and improved renal injury, fetal weight, spiral artery remodeling and placental blood flow in DEX-treated rats. DEX increased SUMOylation in HTR8 and BeWo cells. TAK-981 reversed DEX-induced dysfunction in HTR8 and BeWo cells, such as migration, invasion and syncytialization. Mass spectrum analysis of SUMO1 immunoprecipitation coupled with functional validation showed that SUMOylated proteins related to oxidative stress caused by DEX were reversed by TAK-981 in cultured trophoblasts. TAK-981 mitigated placental oxidative stress in DEX-treated rats. GEO database combined with Western blotting showed upregulated SUMOylation in human placentas with PE. Our findings indicate that protein SUMOylation is one of the key events in PE, particularly in that associated with high glucocorticoid exposure. Targeting placental SUMOylation might be a promising therapeutic strategy for PE.

## 1. Introduction

Preeclampsia (PE) is a leading cause of maternal and fetal mortality and morbidity [1]. The risk factors and pathogenesis of PE are complex and remain far from fully elucidated. A number of studies have shown that increased glucocorticoid (GC) exposure resulting from GC administration in pregnant women with allergic or autoimmune diseases also poses a risk for PE [2,3,4,5], and circulatory GC level is increased in PE patients [3,4,6,7]. Our previous studies have demonstrated that exposure to GC during pregnancy causes hallmark features of PE in rats [8], which supports that elevated GC is involved in PE development and progress.

SUMOylation, a post-translational modification, plays crucial roles in cellular processes, and various diseases progress through regulation of protein stability, activity and subcellular localization as well as epigenetic modifications [9]. The SUMOylation cascade involves three core steps. Initially, major SUMO proteins (SUMO1, SUMO2/3) are activated by the E1 enzyme (SAE1/UBA2 heterodimer), subsequently transferred to the E2 enzyme (UBC9), and ultimately conjugated to target proteins via E3 ligases [10]. Notably, SUMOylation is a dynamic and reversible process characterized by the cyclic balance between SUMOylation and deSUMOylation. Specifically, SUMO1, SUMO2/3, or SUMO4 undergo activation, conjugation, and ligation sequentially, and then bind to relevant substrates to promote their SUMOylation. In contrast, deSUMOylation, the reverse process, is mediated by proteases of the SENP (sentrin-specific protease) family, which cleave the SUMO moiety from modified substrates to reverse their SUMOylation status [11,12,13]. The dynamic balance of SUMOylation regulated by these key genes may be involved in the pathogenesis and progression of numerous diseases by affecting specific tissues or cells [14,15,16]. As for preeclampsia (PE), several studies have reported increased SUMOylation in the placentas of PE patients [17,18,19]; other studies have demonstrated that the balance between SENP2 and SUMO2/3 is essential for trophoblast differentiation and function [20,21]. However, whether intervention of SUMOylation processes affects PE progress has not been reported.

As mentioned, our previous study has shown that exposure to dexamethasone (DEX) during pregnancy leads to PE-like features in rats [8]. Some studies have demonstrated that GC can regulate the SUMOylation cascade in some cells [22,23]. Moreover, our data of RNA-seq of placental tissues in pregnant rats exposed to GC showed that protein deSUMOylation pathways are significantly enriched. We therefore hypothesized that protein SUMOylation may be involved in PE progress related to high GC exposure. The purposes of the present study were to explore whether protein SUMOylation contributes to PE development and progress, and to uncover potential therapeutic roles of SUMOylation inhibitors for PE intervention, in particular, in a PE model associated with high GC exposure. First, we examined protein SUMOylation in the placentas of a dexamethasone (DEX)-induced PE rat model and found that SUMOylation was elevated in the placental tissues of this PE rat model. TAK-981 is a potent and highly selective small-molecule inhibitor, which directly targets the SUMO-activating enzyme E1—the rate-limiting enzyme initiating the SUMOylation cascade [24]. By acting on this key upstream target, TAK-981 specifically blocks the initiation step of the entire SUMOylation process with high specificity [24,25]. Therefore, we then investigated the effects of TAK-981 on PE features of DEX-induced PE rat models and trophoblast function in cultured human trophoblast HTR8 cells and BeWo cells with DEX treatment. We then elucidated the potential mechanisms underlying SUMOylation caused by DEX in cultured cells. Finaly, we examined protein SUMOylation occurrence in human placentas with PE. The results of the present study would provide new insights into the pathogenesis of PE and its related interventional strategies.

## 2. Materials and Methods

### 2.1. Human Samples

All ethical approval was obtained from the Ethical Committee of Medical Research, Xiangya Hospital, Central South University, Changsha, China (Approval Number: Keshen 2024030351). Informed written consent was provided by all participants prior to sample collection. Placental tissues were harvested from normotensive pregnant women at term gestation (*n* = 18; gestational age: 37–40 weeks) and patients with preeclampsia (PE, *n* = 18; gestational age: 36–40 weeks) who underwent elective cesarean section without labor initiation. PE was defined as pregnancy-induced hypertension (systolic/diastolic blood pressure ≥140/90 mm Hg) accompanied by proteinuria (≥0.3 g/24 h) [1], in women with no history of hypertension or other pre-existing medical conditions before pregnancy, such as type 1/2 diabetes mellitus, kidney disease, liver disease, autoimmune disease, etc. Moreover, pregnant women with multiple pregnancies, fetal growth restriction and intrahepatic cholestasis of pregnancy as well as those with gestational diabetes mellitus were excluded. The healthy pregnant women with singleton pregnancy and without pre-existing diseases were rerolled as controls. The clinical characteristics of the included pregnant women are summarized in Table S1.

All placental samples were harvested within 30 min after cesarean section to minimize tissue degradation. The villous tissues (1 cm × 1 cm × 1 cm) were collected about 2 cm away from the root of umbilical cord. Fresh samples were immediately snap-frozen in liquid nitrogen and stored at −80 °C for subsequent protein and RNA extraction.

### 2.2. Animals

All experimental protocols were approved by the Experimental Animal Ethics Committee of Xiangya Hospital, Central South University (Approval Number: Dongwulunshen2024030351). Sprague–Dawley rats aged 8–10 weeks were purchased from the SLAC Laboratory Animal Company (Changsha, China). Animals were housed under controlled conditions of 55% relative humidity, 25 °C ambient temperature, and a 12 h light/dark cycle. Female and male rats were mated overnight, followed by vaginal smears the next morning. Gestation day (GD) 0.5 was designated when massive spermatozoa were detected in the smears. From GD7.5 to GD17.5, pregnant rats (dams) received a subcutaneous (s.c.) injection of dexamethasone-21-phosphate disodium salt (DEX, Sigma-Aldrich, St. Louis, MO, USA) at a concentration of 0.39 mg/mL (equivalent to 0.1 mg/kg body weight) once daily, while the control group received an equal volume of saline. Additionally, another cohort of pregnant rats was administered a combination treatment of DEX and TAK-981 (MedChemExpress, Shanghai, China) via intraperitoneal (i.p.) injection at 1 mg/kg once daily from GD12.5 to GD16.5. The dosage of DEX was determined based on our preliminary study; in both our prior research and the present study, the incidence of PE-like symptoms in pregnant rats reached 100% [8]. The TAK-981 dosage was optimized in our preliminary study. As shown in Figure S1, intraperitoneal injection (i.p.) of high-concentration TAK-981 (7.5 mg/kg, once every two days) of TAK-981 [23] resulted in pregnancy loss and fetal growth restriction (FGR). Meanwhile, injection of TAK981 at 1 mg/kg/d [26] from GD12.5 to GD16.5 did not affect litter size and fetal weight. Rat blood pressure was measured as described previously [8]. Placental blood flow was assessed using Doppler ultrasonography on GD18.5. From GD19 to GD20, the pregnant rats were placed in metabolic cages for a 24 h acclimation period to minimize stress-related confounding factors. Subsequently, from GD20 to GD 20.5, the rats were subjected to food deprivation with free access to water, and 12 h urine samples were collected continuously using the metabolic cages. On GD20.5, maternal blood samples were collected prior to euthanasia, followed by tissue harvesting. For euthanasia, rats were placed in a sealed anesthesia chamber by MATRX Small Animal-Specific Inhalation Anesthesia Machine VMR (Midmark, Dayton, OH, USA), where 5–7% isoflurane mixed with pure oxygen (oxygen flow rate: 2 L/min) was administered. The procedure continued until the disappearance of the corneal reflex and respiratory movement was confirmed; thereafter, isoflurane ventilation was maintained for an additional 5–10 min to ensure irreversible death.

The sample size was determined by using the resource equation method, which is widely accepted for exploratory animal studies in biomedical research [27,28], according to the formula E = N − K, where N = K × *n*, which can be rearranged to *n* = E/K + 1. Here, E (error degrees of freedom) is recommended to range from 10 to 20, and K = 3 (number of groups). The calculated appropriate sample size per group was 5–8 animals, and we therefore set *n* = 5 per group in the present study.

Regarding the assessment of fetal and placental weights, the specific and standardized methodology was detailed as follows: After euthanasia of pregnant rats on GD20.5, uterine horns were carefully dissected, and fetuses with attached placentas were separated gently. For fetal weight measurement, residual placental membranes and amniotic fluid were completely removed from each fetus, and the fetal surface was blotted dry. For placental weight measurement, the placenta was detached from the fetal umbilical cord, and adherent connective tissues were trimmed off meticulously before being blotted dry. Subsequently, the weight of each individual fetus and corresponding placenta was accurately quantified using an electronic analytical balance with a precision of 0.001 g (ILIN, Shanghai, China), and all weight data were recorded systematically for subsequent statistical analysis.

### 2.3. Transcriptome Sequencing and Gene Set Enrichment Analysis (GSEA) Analysis

RNA-sequencing was performed by Novogene Co., Ltd. (Beijing, China). Briefly, FPKM of each gene was calculated based on the length of the gene and reads count mapped to this gene. Transcriptomic analysis was performed using GSEA with all expressed genes on the NovoMagic “https://magic.novogene.com/ (accessed on 7 April 2026)”. FDR correction was applied for multiple testing, and FDR < 0.25 was used as the threshold for significant enrichment, consistent with standard GSEA practice [29].

### 2.4. Total RNA Extraction and Quantitative Real-Time PCR (q-PCR)

RNAex Pro reagent (AGBIO, Changsha, China) was used to extract total RNAs from the frozen tissues. In total, 1 µg RNA was reverse transcribed to generate cDNA by using PrimeScript RT Master Mix Kit (TaKaRa Bio, Shiga, Japan). The primers used for Q-PCR were synthesized by Tsingke Biotechnology (Beijing, China). Q-PCR reaction was carried out on MiniOpticon™ Real-Time PCR Detection System (BioRad, Hercules, CA, USA). The reaction solution consisted of 2.0 μL diluted cDNA, 0.2 μM of each paired primer and 1 × ChamQ Universal SYBR qPCR Master Mix (Vazyme Biotechnology, Nanjing, China). The housekeeping gene β-actin was used as an internal control. The specificity of PCR products was examined by the melting curve at the end of the amplification and subsequent sequencing. To determine the relative quantitation of gene expression for both target and housekeeping genes, the comparative Ct (threshold cycle) method with arithmetic formulae (2^−ΔΔCt^) was used. All primer sequences are listed in Table S3.

### 2.5. Western Blotting Analysis

For protein extraction, 30 mg of tissues were homogenized in ice-cold RIPA (Beyotime, Shanghai, China) with 1% proteinase inhibitor solution PMSF (Beyotime) and 1% phosphatase inhibitor cocktail (Thermo Scientific, Waltham, MA, USA) followed by centrifugation at 12,000 rpm and 4 °C for 30 min. Protein concentrations in the lysates were quantified using the BCA Protein Assay Kit (Ecotopbio, Guangzhou, China). Equal amounts of 30 μg protein per lane were loaded for separation with SDS-PAGE gels (Epizyme Biotech, Shanghai, China) and transferred to PVDF membranes (Merck-Millipore, Billerica, MA, USA) followed by overnight incubation with primary antibodies against target proteins and β-actin (used as the internal reference), which are listed in Supplementary Table S2. The membrane was washed with 0.1% Tween 20 in TBS and incubated with a goat anti-rabbit or mouse IgG antibody (Beyotime) for 1 h at room temperature. Protein bands were detected using ECL Western blotting Substrate (Ecotopbio). In some cases, we analyzed gray values of protein bands using ImageJ version 1.54d.

### 2.6. Cell Culture

HTR8 is an extravillous trophoblast (EVT) cell line derived from human first-trimester placenta (a generous gift from Prof. Charles H. Graham, Queen’s University, Kingston, ON, Canada). The cells were maintained in DMEM (Boster Biological Technology, Wuhan, China) containing 10% fetal calf serum (Thermo Scientific), penicillin (100 U/mL) (Thermo Scientific) and streptomycin (100 mg/mL) (Thermo Scientific) at 37 °C in 5% CO_2_–95% air humidified atmosphere.

BeWo cells purchased from Shanghai Jinyuan Biotechnology Co., Ltd. (Shanghai, China) were cultured and maintained in Ham’s F12K (Jinyuan Biotechnology) containing 10% fetal calf serum, penicillin (100 U/mL) and streptomycin (100 mg/mL) at 37 °C in 5% CO_2_–95% air humidified atmosphere. Forskolin (MedChemExpress) was dissolved in DMSO and stored at −20 °C until use. Following cell attachment, BeWo cells were treated with FSK for 48 h.

### 2.7. Migration and Invasion Assay

Cell migration of HTR8 cells was evaluated via the scratch wound healing assay. Briefly, adherent cells were seeded in 6-well plates. Uniform scratches were created across the cell monolayer using a sterile 200 μL pipette tip. Detached cells were removed by washing with pre-warmed PBS, followed by the addition of serum-free medium. Images of the scratched areas were captured at 0 h and 24 h using an inverted microscope. The wound closure rate was calculated by measuring scratch widths with ImageJ software, with three independent replicates per group.

Invasion function of HTR8 cells was measured using 24-well BD Matrigel invasion chambers (BD Biosciences, San Jose, CA, USA). Briefly, cells were seeded into the top chamber and were allowed to migrate toward the bottom chamber. After 24 h incubation, the underside of the membrane was stained with DAPI (Servicebio, Wuhan, China). The number of the cells on the membrane from 3 random fields at ×200 magnification was counted under the microscope.

### 2.8. Measurement of Blood Pressure

Blood pressure was monitored by a noninvasive tail-cuff system (BP-300A automatic noninvasive blood pressure measurement system, Techmen (Taoyuan, Taiwan)) from GD13.5 to 17.5 every two days.

### 2.9. Analysis of Microalbumin Concentration in Urine

Random, single-void urine specimens were obtained individually in a metabolic cage on GD20–20.5 under fasting conditions, then used to determine microalbumin by a microalbumin assay kit (HOMA Biological, Beijing, China).

### 2.10. Histological Morphology of Kidney

For glomerular pathological assessment, 3 µm kidney paraffin sections were deparaffinized in xylene and rehydrated through a graded ethanol series, followed by Hematoxylin and Eosin (H&E) staining. Pathological evaluation was performed in consultation with an experienced renal pathologist at Xiangya Hospital.

### 2.11. Histological Morphology and Immunostaining of Placenta

For placental histological and immunofluorescence (IF) analysis, 5 µm placenta paraffin sections were deparaffinized in xylene and rehydrated through a graded ethanol series, followed by H&E staining and immunohistochemical staining. Trophoblasts and smooth muscle cells in rat placentas and uteri were identified by IF. Briefly, after blocking with 5% bovine serum albumin (Servicebio, Wuhan, China), sections were incubated overnight at 4 °C with primary antibodies against pan-cytokeratin (pan-CK) (1:500, Dako, Glostrup, Denmark) or α-smooth muscle actin (α-SMA) (1:400, Servicebio). Specific secondary antibodies labeled with Alexa Fluor^®^ 488 or Alexa Fluor^®^ 594 (1:500, Abcam, Cambridge, MA, USA) were utilized. Images were captured with a Nikon microscope (Nikon, Tokyo, Japan).

### 2.12. Measurement of Uteroplacental Blood Flow

Uteroplacental blood flow was monitored by Doppler ultrasonography on GD18.5. Pregnant rats were first placed in an anesthesia induction chamber and administered 4–5% isoflurane in oxygen. Once anesthetized, the rats were transferred to the operating platform, where anesthesia was maintained with 1.5–2.5% isoflurane delivered continuously via a face mask throughout the entire procedure, and abdominal hair was removed using a depilatory agent. A high-frequency 22 MHz linear array probe (Vevo 2100, FUJIFILM VisualSonics, Toronto, ON, Canada) was used to acquire Doppler waveforms and analyze peak systolic velocity (PSV). For each rat, 2–4 implantation sites were examined, and 2–3 waveforms were recorded for each of the spiral arteries (SA), maternal canal, and umbilical artery (UmbA) per implantation site.

### 2.13. Immunoprecipitation (IP)

Cells were lysed in IP-specific RIPA buffer (Servicebio), followed by centrifugation at 12,000× *g* for 20 min. Prior to sample incubation, we pre-washed the protein A/G agarose beads (MedChemExpress) three times at 4 °C for 0.5 h with IP lysis buffer to remove contaminants from the manufacturer. All immunoprecipitation reactions were performed in sterile microcentrifuge tubes to avoid potential cross-contamination. Then, overnight incubation was performed at 4 °C with specific antibodies or isotype-matched IgG at a ratio of 1 μg antibody to 1 mg protein. Precipitated proteins were boiled in 1× SDS sample buffer at 100 °C for 10 min and separated by SDS-PAGE. The gel excision procedures were conducted in a sterile laminar flow hood. We used new sterile scalpel blades and disposable sterile centrifuge tubes for sample handling, and the processed samples were then sent to Applied Protein Technology (Shanghai, China) for subsequent mass spectrometry analysis.

### 2.14. 4D Mass Spectrometry Analysis

4D mass spectrometry was performed by Shanghai Applied Protein Technology Co., Ltd. (Shanghai, China). Gel bands containing the target protein complexes were excised and washed with a destaining solution (30% acetonitrile/100 mM ammonium bicarbonate) until transparent. The bands were sequentially treated with 10 mM DTT (37 °C, 60 min) for reduction, 60 mM iodoacetamide (IAA, room temperature, protected from light, 60 min) for alkylation, dehydrated with acetonitrile, and then lyophilized under vacuum. Trypsin solution (2.5–10 ng/μL) was added at an enzyme-to-protein ratio of 1:50, followed by enzymatic digestion at 37 °C for 20 h. The digested solutions were extracted by sonication for 15 min, combined, and lyophilized under vacuum. Peptides were purified and desalted using ZipTipC18 (Millipore) and reconstituted in 0.1% formic acid.

Separation was performed on a nanoElute nanoliquid chromatography system using a C18 column (15 cm × 150 μm, 2 μm) at a flow rate of 300 nL/min with a 30 min gradient elution (5–35–80% acetonitrile). The chromatographically separated peptides were analyzed using a timsTOF Pro mass spectrometer in positive ion mode (1.5 kV) with PASEF mode for data acquisition (cycle time: 0.95 s, dynamic exclusion: 24 s) and a scanning range of *m*/*z* 100–1700. Protein identification and quantification were performed using MaxQuant version 1.6.14.

### 2.15. Reactive Oxygen Species and Total Antioxidant Capacity Assays

Reactive oxygen species (ROS) level was assessed using the Reactive Oxygen Species Detection Kit (Beyotime), and total antioxidant capacity (TAC) was measured by a Total Antioxidant Capacity Detection Kit (ABTS Method) (Beyotime) according to manufacturer instructions, respectively.

### 2.16. Statistical Analysis

GraphPad Prism 9 (GraphPad Software, Inc.) was used for graphical presentation and statistical analysis. Results are expressed as mean ± standard error of the mean (SEM) per group. Statistical analyses were performed as described in the figure legends, with a *p*-value < 0.05 considered statistically significant. Differences between two groups were compared using either the Mann–Whitney U test (for non-parametric data) or unpaired *t*-test (for parametric data), as appropriate. For multiple comparisons among the three groups, one-way analysis of variance (ANOVA) was applied. No samples were excluded from the analyses.

## 3. Results

### 3.1. SUMOylation Is Elevated in Placentas of DEX-Induced PE Rat Model

**Figure 1:**
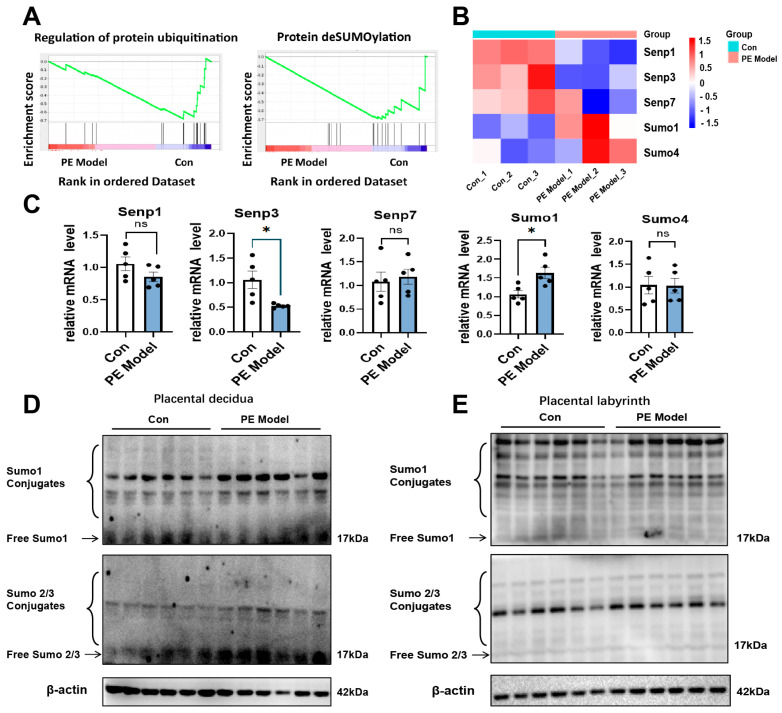
SUMOylation is elevated in placentas of PE model. (**A**) GSEA of the PE model and its control showed the pathways related to regulation of protein ubiquitination and protein deSUMOylation. (**B**) Heatmap of core gene expression profiles of protein deSUMOylation based on the RNA-Seq. (**C**) The relative mRNA levels of core gene of protein deSUMOylation pathways. (**D**,**E**) Protein levels of SUMO1 and SUMO2/3 in placental decidua and (**D**) placental labyrinth (**E**). *n* = 5 in each group. The final lane of each group is a mixed sample that was prepared by mixing equal volumes of protein extracts from multiple individual samples in the group. * *p*  <  0.05, ns: not significant.

Our previous study demonstrated that DEX treatment (0.1 mg/kg) induced PE-like phenotypes in pregnant rats, including increased mean arterial pressure (MAP), systolic blood pressure (SBP), renal injury, and impaired placental blood flow [8]. To characterize the placental changes in these PE models, RNA sequencing (RNA-seq) was performed [8]. Sequencing data are available in the National Genomics Data Center under accession “PRJCA006654”. Through GSEA enrichment analysis, we noted that the regulation of protein ubiquitination and protein deSUMOylation pathways were significantly enriched, and the related core gene heatmap showed that the genes that mediated deSUMOylation were *Senp1*, *Senp3*, *Senp7*, *Sumo1* and *Sumo4* (Figure 1A,B). Subsequently, we confirmed via qPCR that in the PE model group, the expression of *Senp3*, a gene regulating deSUMOylation, was decreased, whereas the expression of *Sumo1*, which mediates SUMOylation, was elevated (Figure 1C). We further examined SUMOylation levels in placentas of the DEX-induced PE model (hereafter referred to as the PE model). As shown in Figure 1D, conjugated SUMO1 was significantly increased and conjugated SUMO2/3 was slightly elevated in placental decidual tissues (containing junction zone of placenta) of the PE model. Additionally, conjugated SUMO1 but not SUMO2/3 was moderately increased in the placental labyrinth (Figure 1E).

### 3.2. SUMOylation Inhibitor TAK-981 Alleviates Hallmark Features of PE in Animal Model

**Figure 2:**
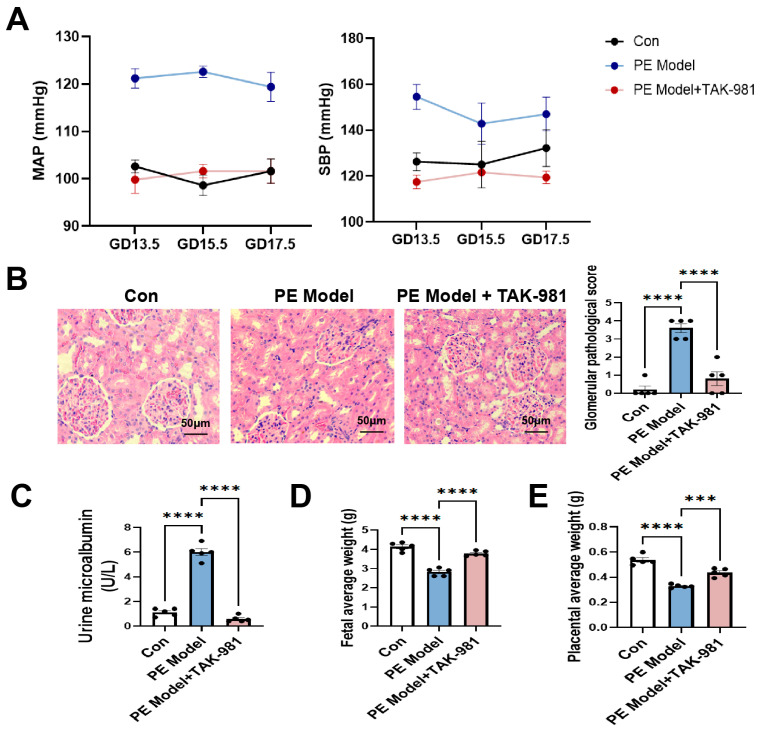
SUMOylation inhibitor TAK-981 alleviates features of PE in DEX-treated rats. (**A**) MAP and SBP were measured from GD13.5, GD15.5 and GD17.5. (**B**) SBP was measured on GD20.5. Error bar, SEM, *n* = 5 in each group. (**B**) Morphology of glomeruli stained by H&E. (**Left panel**): The representative images (400×). (**Right panel**): Histopathological score of glomerular pathology. Error bar, SEM, *n* = 5 in each group. (**C**) Microalbumin (U/L) in urine. Error bar, SEM, *n* = 5 in each group. (**D**,**E**) Fetal (**D**) and placental (**E**) average weight from 5 dams (each group) measured on GD 20.5. It represents mean fetal and placental weight from each dam. Error bar, SEM, *n* = 5 in each group. *** *p*  <  0.001, **** *p*  <  0.0001.

We next explored the therapeutic efficacy of TAK-981, a selective SUMOylation inhibitor, in a DEX-induced PE model. As shown in Figure 2, TAK-981 treatment significantly reversed the elevated blood pressure in the PE model, and it also reduced urinary protein in the PE model and improved its renal injury score (Figure 2A–C). Additionally, TAK-981 restored fetal and placental weights in the PE model to near-normal levels (Figure 2D,E).

### 3.3. TAK-981 Improves Impaired SA Remodeling, Uteroplacental Blood Flow and Suppresses Placental SUMOylation

**Figure 3:**
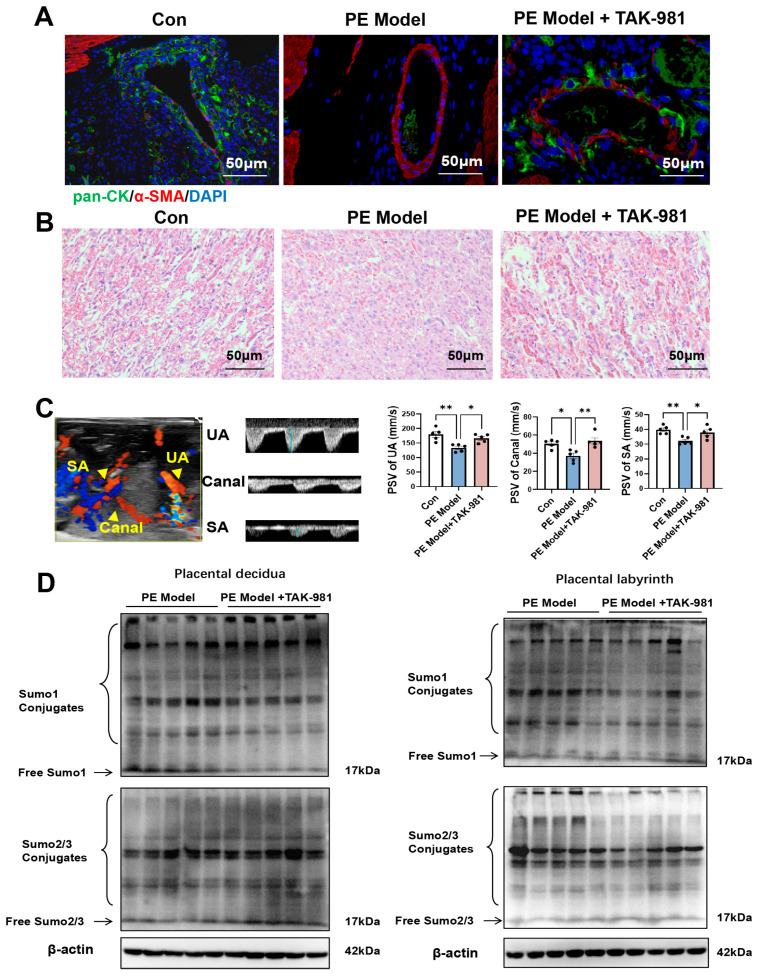
TAK-981 improves impaired SA remodeling, uteroplacental blood flow and reduces placental SUMOylation. (**A**) Placental SA remodeling. Representative images of pan-CK and α-Sma IF staining in SA (400×). (**B**) Placental morphology. Representative H&E images of labyrinth zone (400×). (**C**) Doppler ultrasonography. (**Left panel**): The representative images of SA, canal and fetal UA visualized by ultrasound biomicroscopy. (**Right panel**): Cumulative data of the PSV of SA, Canal and UA. Error bar, SEM, *n* = 5 in each group. (**D**) Protein levels of conjugated SUMO1 and SUMO2/3 in placental decidua and placental labyrinth. Error bar, SEM, *n* = 5 in each group. * *p*  <  0.05, ** *p*  <  0.01.

Normal placentation relies on proper spiral artery (SA) remodeling [30]. Our prior work identified SA remodeling disorders in PE models, characterized by increased α-SMA staining intensity and reduced trophoblast infiltration in SA [8]. TAK-981 treatment reversed this pathological phenotype, as evidenced by increased cytokeratin (CK) expression and decreased α-SMA staining in SA (Figure 3A). Furthermore, TAK-981 enhanced the complexity of the labyrinth vascular network, significantly improved peak systolic velocity (PSV) in SA, maternal central channels (Canal), and umbilical arteries (UA), so as to restore uteroplacental blood flow (Figure 3B,C). Notably, TAK-981 abrogated the elevation of SUMOylation, in particular, conjugated SUMO1 level, in the decidua and labyrinth regions of the placentas in the PE model (Figure 3D). For conjugated SUMO2/3 level, TAK-981 treatment could slightly reduce its level in the decidual region and robustly reduced its level in the labyrinth region in the PE model.

### 3.4. SUMOylation Is Elevated in Cultured Trophoblasts Treated with DEX

**Figure 4:**
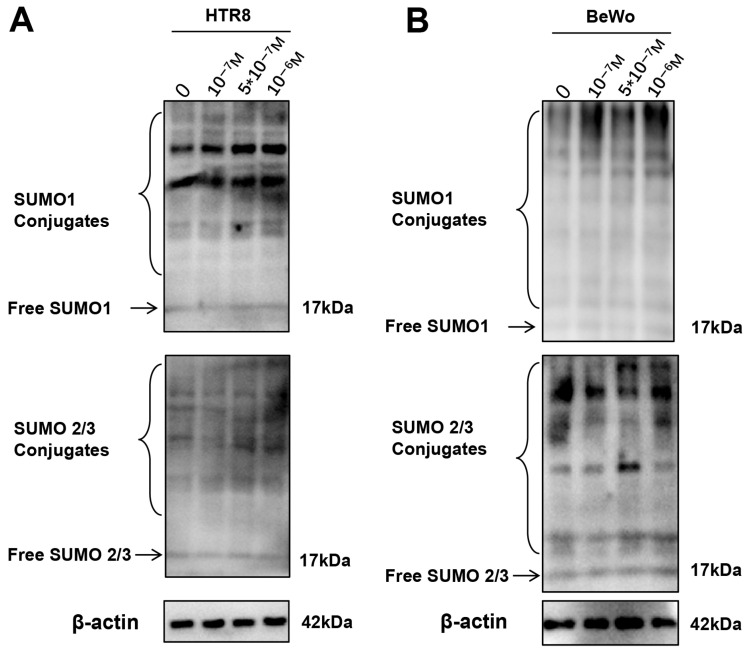
SUMOylation is elevated in cultured trophoblasts treated with DEX. HTR8 and BeWo cells were treated with DEX (10^−7^ M), DEX (5 × 10^−7^ M), DEX (10^−6^ M) for 24 h. The cells were then harvested for WB. (**A**,**B**) Protein levels of SUMO1 and SUMO2/3 in HTR8 cells (**A**) and BeWo cells (**B**).

Trophoblasts are critical cellular components of the placenta, and normal placental development and function depend on the proper differentiation of decidual/junctional invasive trophoblasts and syncytialization of labyrinthine trophoblasts. Dysfunction of these processes contributes to PE and other pregnancy-related complications [31,32,33]. Previous studies have reported that SUMOylation exhibits tissue- and cell-type specificity [34]. To further characterize SUMOylation changes in invasive trophoblasts and syncytiotrophoblasts following DEX treatment, we utilized two in vitro models: the human EVT cell line HTR8 (a well-established model for invasive trophoblasts) and forskolin-induced syncytialization of the human trophoblast cell line BeWo [35,36]. As shown in Figure 4, DEX treatment at various concentrations significantly increased conjugated SUMO1 levels in HTR8 cells, and DEX (5 × 10^−7^ M, 10^−6^ M) caused an increase in SUMO2/3 conjugation in HTR8 cells (Figure 4A). In BeWo cells, DEX treatment significantly upregulated conjugated SUMO1 but had no significant effect on SUMO2/3 levels (Figure 4B). These in vitro findings are consistent with the SUMOylation patterns observed in decidual and labyrinthine tissues of the in vivo PE model, further supporting a role for SUMOylation dysregulation in trophoblast dysfunction under PE-like conditions.

### 3.5. SUMOylation Inhibitor Alleviates DEX-Induced Trophoblast Dysfunction

**Figure 5:**
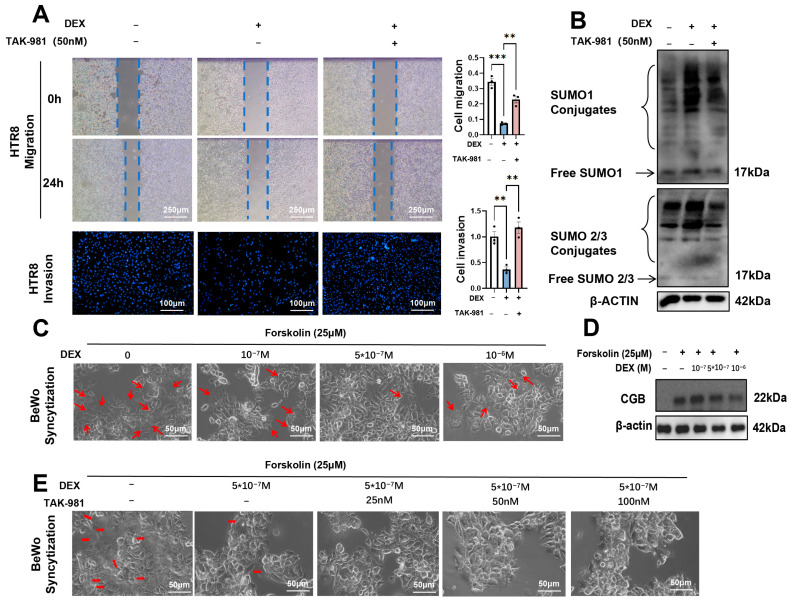
SUMOylation inhibitor alleviates DEX-induced trophoblast dysfunction. HTR8 cells were treated with DEX (5 × 10^−7^ M), or combination with TAK-981 (50 nM) for 24 h. The cells were then used for the migration and invasion analysis. In some cases, cells were harvested for WB analysis. BeWo cells were treated with forskolin (25 μM) for 48 h; meanwhile, they were treated with DEX (10^−7^ M), DEX (5 × 10^−7^ M), DEX (10^−6^ M), or combination with TAK-981 (50 nM) for 48 h. The cells were then used for the syncytialization analysis. In some cases, cells were harvested for WB analysis. (**A**) Analysis of the migration and invasion function in HTR8 cells. (**Left panel**): Representative images of scratch wound healing assay (40×) and cells moved to the membrane underside (100×). (**Right panel**): Analysis of cumulative cell migration and invasion capacity. Blue dashed lines indicate the leading edges of cell migration.Error bar, SEM, *n* = 3 in each group. (**B**) Protein levels of SUMO1 and SUMO2/3 in HTR8 cells. (**C**) Representative syncytialization images of BeWo cells. Red arrows indicate cells that have undergone syncytialization. (**D**) Protein levels of CGB (quantifiable syncytization) in BeWo cells. (**E**) Representative syncytialization images of BeWo cells. Red arrows indicate cells that have undergone syncytialization. ** *p*  <  0.01, *** *p*  <  0.001.

Our previous study demonstrated that DEX (5 × 10^−7^ M) caused HTR8 dysfunction, which was manifested as reduced migration and invasion ability [8]. As described above, DEX treatment resulted in elevated SUMOylation levels in both HTR8 and BeWo cells. Prompted by this observation, we hypothesized that reducing SUMOylation might ameliorate DEX-induced dysfunction in these trophoblast cell lines. We first validated that TAK-981 (25 nM, 50 nM, 100 nM) [24] effectively suppressed cellular SUMOylation (Figure S2A,B). We further investigated the impact of TAK-981 on DEX-induced trophoblast impairment. As shown in Figure 5, TAK-981 (50 nM) significantly attenuated the DEX (5 × 10^−7^ M)-induced upregulation of SUMOylation and restored the impaired migratory and invasive capacities of HTR8 cells (Figure 5A,B). In the present study, we also found that DEX disrupts BeWo cell function. As illustrated in Figure S3A,B, forskolin (25 nM, 50 nM, 100 nM) consistently induced syncytialization in BeWo cells. However, TAK-981 (25 nM, 50 nM, 100 nM) failed to significantly ameliorate the DEX-induced abnormal syncytialization of BeWo cells (Figure 5C–E).

### 3.6. SUMOylation Inhibitor Improves Abnormal Expression of the Factors Linked to Oxidative Phosphorylation Caused by DEX in Cultured Trophoblasts and Mitigates DEX-Induced Oxidative Stress in Cultured Trophoblasts and Placentas of DEX-Treated Rats

**Figure 6:**
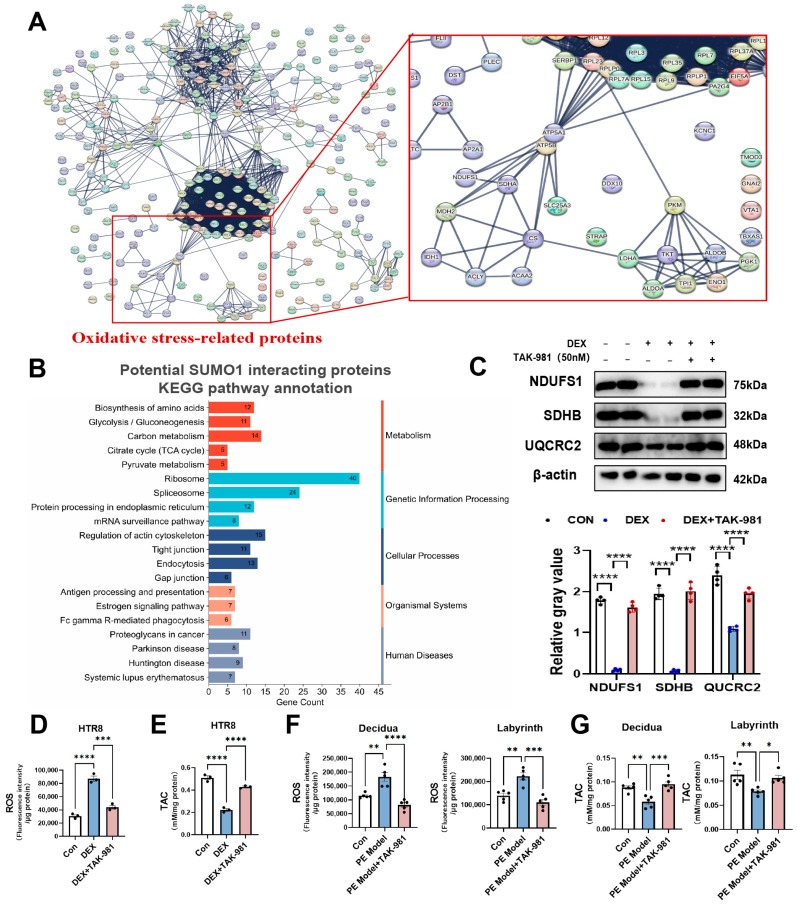
SUMOylation mitigates oxidative stress of placenta and trophoblast. (**A**) PPI network of potential SUMO1-interacting proteins in HTR8 cells. The PPI network was constructed using the STRING database with high confidence (interaction score > 0.9). Only the key focused components are displayed in the figure due to the complexity and large scale of the full network. (**B**) KEGG enrichment analysis of potential SUMO1-interacting proteins in HTR8 cells. (**C**) Protein levels of NDUFS1, SDHB, UQCRC2 in HTR8 cells. (**Upper panel**): representative blotting images. (**Lower panel**): analysis of cumulative data of proteins’ relative gray value. Error bar, SEM, *n* = 4 in each group. (**D**,**E**) Oxidative stress states in HTR8 cells. ROS (**D**) and TAC (**E**) levels. Error bar, SEM, *n* = 4 in each group. (**F**,**G**) Oxidative stress state of placental decidua and labyrinth in animal model. ROS (**F**) and TAC (**G**) levels. Error bar, SEM, *n* = 5 in each group. * *p*  <  0.05, ** *p*  <  0.01, *** *p*  <  0.001, **** *p*  <  0.0001.

To identify potential specific interacting proteins with SUMO1, we performed SUMO1 immunoprecipitation (IP) on lysates of DEX-treated HTR8 cells, followed by mass spectrometry analyses. A total of 299 potential SUMO1-interacting proteins were identified. Protein–protein interaction (PPI) analysis indicated the presence of numerous oxidative stress-associated proteins such as NDUFS1, SDHA, etc. KEGG enrichment analysis further revealed these proteins were significantly enriched in oxidative stress-related metabolic pathways, including glycolysis, the tricarboxylic acid (TCA) cycle, and carbon metabolism [37,38,39] (Figure 6A,B). In addition, we found that DEX significantly down-regulated the levels of oxidative phosphorylation related proteins such as NDUFS1, SDHB and UQCRC2 in HTR8 cells, and TAK-981 reversed this effect (Figure 6C). Based on these findings, we investigated oxidative stress changes in DEX-treated HTR8 cells. DEX treatment induced a significant increase in reactive oxygen species (ROS) levels and a reduction in total antioxidant capacity (TAC), both of which were significantly abrogated by TAK-981 treatment (Figure 6D,E). Moreover, TAK-981 ameliorated placental oxidative stress in the PE model by decreasing ROS accumulation and enhancing placental TAC (Figure 6F,G). Collectively, our results suggest that TAK-981 may ameliorate trophoblast dysfunction and PE-like symptoms through suppressing oxidative stress in both placenta and trophoblasts.

### 3.7. SUMOylation Is Elevated in Placentas of PE Patients

**Figure 7:**
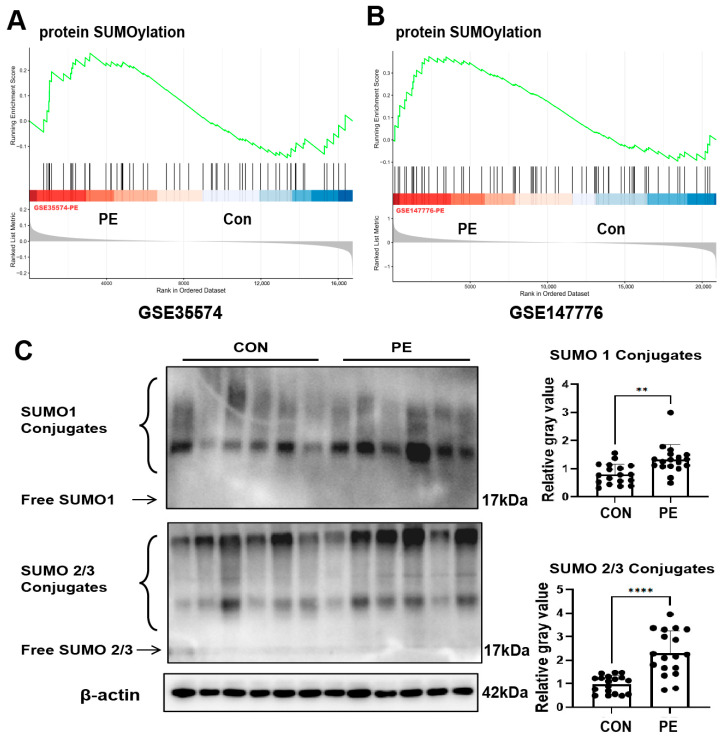
SUMOylation is elevated in placentas of PE patients. (**A**,**B**) GSEA of the datasets GSE35574 (**A**) and GSE147776 (**B**) showed the pathway related to protein SUMOylation. (**C**) Protein levels of SUMO1 and SUMO2/3. (**Left panel**): Representative blotting images. (**Right panel**): Analysis of cumulative data of SUMO1 and SUMO2/3 conjugates protein relative gray value. Error bar, SEM, *n* = 18 in each group. ** *p*  <  0.01, **** *p*  <  0.0001. CON: control; PE: preeclampsia.

As mentioned earlier, several studies have reported increased SUMOylation in placentas of PE patients [17,18,19]. We further analyzed the changes in SUMOylation in the placentas of PE patients in the GEO database. As shown in Figure 7A, in the GSE35574 dataset, GSEA enrichment analysis revealed that protein SUMOylation in placentas of PE patients was enriched compared with the control group (CON). Similarly, in the GSE147776 dataset, compared with CON, placental protein SUMOylation of PE patients was significantly enriched (Figure 7B). Subsequently, we conducted WB on the placentas of PE patients (*n* = 18) and healthy control (CON) pregnant women (*n* = 18). The results showed that the levels of SUMO1 and SUMO2/3 in the placentas of PE were significantly increased compared to controls (Figure 7C).

## 4. Discussion

In the present study, we demonstrated the upregulation of the SUMOylation pathway in the placentas of DEX-treated rats by RNA-seq analysis and then confirmed that conjugated SUMO1 and SUMO2/3 levels were elevated in placental tissues in this rat model. The selective SUMOylation inhibitor TAK-981 robustly alleviated hallmark features of PE in DEX-treated rats. Using cultured cell models, we revealed that DEX treatment promoted conjugated SUMO1 and SUMO2/3 levels in HTR8 and BeWo cells, and TAK-981 robustly ameliorated DEX-induced dysfunction in HTR8 and BeWo cells. These data indicate that SUMOylation plays an important role in the pathogenesis of PE related to high GC exposure.

As mentioned, a number of studies have implicated that high GC exposure may directly or indirectly impact the pathogenesis of PE. For instance, various adverse factors during pregnancy, such as psychological stress and excessive alcohol consumption, can induce elevated GC levels and subsequently contribute to PE development [40,41]. Additionally, increased GC exposure resulting from glucocorticoid administration in pregnant women with allergic or autoimmune diseases also poses a risk for PE [2,3,4,5]. Numerous studies have documented elevated levels of active GCs in the maternal and umbilical cord circulation of PE patients [2,6,42]. Both human and rodent placental development belong to the hemochorial placentation and SA remodeling is a key event for normal placentation [43]. Therefore, the pregnant rodent model is implicated to be a good model for the study of PE pathogenesis. Our previous studies have demonstrated that high GC exposure could lead to hallmark features of PE in pregnant rats [8],which supports the importance of a high GC level in PE progress. In this PE model, we found elevated SUMOylation in the decidual region (containing the junction zone of placenta) and the labyrinth region. In rodents, placentas consist of a junction zone and a labyrinth zone. The junction zone is tightly connected to decidua. There are several cell types in the junction zone, such as spongiotrophoblast (SpT), glycogen trophoblasts (Gly-T) and spiral artery-associated trophoblast giant cells (SpA-TGCs). These trophoblasts could invade into decidua and cause SA remodeling, a critical event during placentation [44,45]. It is known that HTR8 cells belong to extravillous trophoblasts (EVTs) in humans. EVTs are the trophoblasts in SA remodeling in human placentation [30]. Moreover, impaired SA remodeling is believed to play important roles in PE pathogenesis. As consistent with the findings in DEX-treated pregnant rats, elevated SUMOylation occurred upon DEX treatment in cultured trophoblasts. Importance, TAK-981 robustly improved PE features and SA remodeling in a DEX-induced PE model, and it also reversed DEX-induced impaired migration and invasion in HTR8 cells. The labyrinth zone in rodent placenta contains syncytiotrophoblasts and sinus TGCs [46]. Our results showed that DEX treatment resulted in elevated SUMOylation in the labyrinth zone of rat placentas, while in cultured BeWo cells, DEX treatment also increased SUMOylation. TAK-981 robustly improved the histology of the labyrinth zone in the DEX-induced PE model and improved syncytialization in BeWo cells. Together, our study provided strong evidence that SUMOylation plays important roles in the development and progression of PE, at least in PE patients associated with high GC exposure.

Oxidative stress is a well-established contributor to PE pathogenesis, as placental oxidative damage impairs trophoblast function, SA remodeling, and uteroplacental hemodynamics [47,48,49,50]. Our previous study has demonstrated that DEX treatment results in mitochondrial dysfunction and oxidative stress in placental tissues in pregnant rats and cultured HTR8 cells, and antioxidant MitoTEMPO reverses DEX-induced PE features in pregnant rats [8], which confirms the importance of oxidative stress in DEX-induced PE development. The present study showed that SUMOylation inhibitor suppressed DEX-induced oxidative stress of placental tissues in pregnant rats and it also reversed DEX-induced oxidative stress in HTR8 cells. These data indicate that oxidative stress occurrence in placentas caused by DEX is associated with SUMOylation. Interestingly, the data of MS demonstrated that many SUMOylated proteins belong to mitochondrial proteins. It is known that some SUMOylated proteins would reduce their stability and cause ubiquitination-mediated degradation [26,51]. Consistently, we showed that a SUMOylation inhibitor reversed DEX-induced reduced NDUFS1, SDHB and UQCRC2 protein levels in HTR8 cells. Together, it indicates that DEX-induced SUMOylation of mitochondrial proteins contributes to mitochondrial dysfunction in trophoblasts. Although a few studies have reported that SUMOylation regulates oxidative stress responses in some tissues [52,53,54], its role in the placenta has not been elucidated. Our study implies that the SUMOylation–oxidative stress axis might contribute to PE progress. It should be pointed out that many proteins were potentially SUMOylated, which were associated with metabolism, cell junction, cytoskeleton, etc., in HTR8 cells after DEX treatment. It indicates that DEX affects various functions of EVTs via SUMOylation of many proteins. Thus, it would be of interest to elucidate the functional changes in these SUMOylated proteins in our future studies.

Some studies have reported increased SUMOylation in the placentas of PE patients [17,18,19]. Consistently, we showed that conjugated SUMO1 and SUMO2/3 levels were elevated in PE placental tissues in humans. Thus, it suggests a potential link between SUMOylation dysregulation and PE pathogenesis. However, PE is a complex and multifactorial pregnancy complication. There are several classifications for PE. For instance, (1) temporal subtypes, such as early-onset preeclampsia (EoPE) and late-onset preeclampsia (LoPE) [55]; (2) phenotypic subtypes by pathophysiology, such as the placental-dominant subtype which is characterized by inadequate placental remodeling, abnormal placentation, and placental ischemia as primary drivers, the maternal vascular/at-risk-dominant subtype with characteristics of maternal comorbidities (e.g., obesity, insulin resistance, hypertension) and exaggerated maternal inflammatory/endothelial responses contributing substantially and mixed/overlap, i.e., where both placental and maternal factors contribute substantially, with variable timing and severity [56]. Of note, SUMOylated proteins were not fully consistent in human placentas and the placentas of the DEX-induced rat PE model, i.e., increased total conjugated SUMO2/3 level in humans but an unchanged level in rat placentas. Such discrepancy might be due to different species, but it might also be attributed to specific PE animal models, i.e., a PE model caused by high GC exposure. In fact, high GC exposure is one of risk factors for PE. Thus, it would be of importance to define the characteristics of protein SUMOylation in the placentas from the patients with different PE subtypes or different etiologies of PE, thereby confirming the importance of SUMOylation in PE development and gaining deep insight into PE pathogenesis.

TAK-981 has shown efficacy in preclinical models of acute myeloid leukemia and liver fibrosis [25,57,58]; its potential in PE or other pregnancy-related disorders has not been explored. Our study thus represents the first exploratory attempt to apply a SUMOylation inhibitor during pregnancy. We showed that TAK-981 at low-dose (1 mg/kg/d) from GD12.5–16.5 did not cause obvious adverse pregnancy outcomes, but it robustly ameliorated DEX-induced PE features in pregnant rats. Thus, it would be of interest to further investigate whether TAK-981 is a potential reagent for PE treatment. However, it should be noted that TAK-981 could affect the immune function in several preclinical models [24,59,60]. Moreover, it is well known that GCs modulate immune function [61,62]. Dysfunction of the immune system might be involved in PE development caused by high GC exposure. Thus, the effects of TAK-981 on the immune system might contribute to the beneficial effects of TAK-981- on DEX-induced PE progression. In addition, TAK-981 is a global SUMO E1 inhibitor; its immunological effects and long-term impacts on both mothers and fetuses also require evaluation. Nevertheless, future in vivo studies regarding the roles of TAK-981 in PE pathogenesis should be studied in multiple PE animal models, and immunological parameters and long-term safety should also be closely monitored.

There are several limitations in present study. For instance, (1) we just investigated the role of TAK-981 in a DEX-induced PE model. It would be of interest to study whether TAK-981 has a therapeutic role in other PE models such as eNOS-related PE models; (2) it is known that the etiology and pathogenesis of patients with PE exhibit significant heterogeneity. The characteristics of placental SUMOylation in PE patients with different etiology or patients with different PE subtypes should be investigated in a large-scale study of human placental samples from multiple centers; (3) we just identified SUMOylated protein linked to SUMO1 by using MS. The SUMOylated proteins linked to SUMO2/3 were not identified in HTR8 trophoblasts. In addition, the SUMOylated protein in BeWo cells were not identified. It is important to understand the SUMOylated proteins in these two cells, thereby indicating the roles of SUMOylation in PE pathogenesis; (4) although our data indicate that mitochondrial proteins such as NDUFS1 and SDHB are SUMOylated upon DEX treatment, the specific sites of SUMOylation in these proteins have not been identified. The elucidation of the specific sites of SUMOylation in these proteins would provide strong evidence for the roles of SUMOylation in oxidative stress occurrence in PE pathogenesis; (5) our data only support proof-of-concept evidence for the beneficial effects of TAK981. Further comprehensive studies investigating the impacts of TAK-981 on pregnancy outcomes, together with long-term fetal and immunological evaluations, will be required to facilitate clinical translation.

## 5. Conclusions

The present study has demonstrated significant upregulation of conjugated SUMO1 and SUMO2/3 levels in placental tissues in a DEX-induced animal PE model. In human placental tissues, conjugated SUMO1 and SUMO2/3 levels were also increased in PE patients. The selective SUMOylation inhibitor TAK-981 robustly alleviated hallmark features of PE in DEX-treated pregnant rats. We revealed that DEX treatment promoted conjugated SUMO1 and SUMO2/3 levels in HTR8 and BeWo cells, and TAK-981 robustly ameliorated DEX-induced dysfunction in HTR8 and BeWo cells, which supports the importance of SUMOylation in PE development caused by high GC exposure. Mechanically, we revealed that SUMOylation occurred in mitochondrial proteins such as NDUFS1, SDHB and UQCRC2, which might contribute to oxidative stress in placentas induced by DEX. Our data highlight that targeting placental SUMOylation might be a potential therapeutic strategy for PE, in particular, the PE related to high GC exposure. Further mechanistic and translational studies are warranted in the future.

## Data Availability

The RNA-seq data generated in this study have been deposited in the National Genomics Data Center “https://ngdc.cncb.ac.cn/ (accessed on 7 April 2026)” under accession number “PRJCA006654”. All other data are contained within the article and Supplementary Material. Any other data employed in this study, both utilized and analyzed, are available upon a request to the corresponding author.

## Supplementary Materials

The following supporting information can be downloaded at https://www.mdpi.com/article/10.3390/antiox15040478/s1, Table S1: Clinical characteristics of the pregnant woman enrolled in this study; Table S2: Antibodies for WB, IF and IP; Table S3: Primers sequence for q-PCR; Figure S1: The Effects of different doses of the SUMO Inhibitor TAK-981 on litter size, fetal weight and placental weight; Figure S2: Sumo inhibitor effectively suppressed cellular SUMOylation; Figure S3: Forskolin induced syncytialization in BeWo cells.

## Abbreviations

The following abbreviations are used in this manuscript:

α-SMA	α-Smooth muscle actin
ANOVA	Analysis of variance
Canal	Maternal central channels
CON	Control
DEX	Dexamethasone
EoPE	Early-onset preeclampsia
EVT	Extravillous trophoblast
FGR	Fetal growth restriction
GC	Glucocorticoid
GD	Gestation day
H&E	Hematoxylin and Eosin
IF	Immunofluorescence
IP	Immunoprecipitation
LoPE	Late-onset preeclampsia
MAP	Mean arterial pressure
pan-CK	pan-cytokeratin
PE	Preeclampsia
PPI	Protein–protein interaction
PSV	Peak systolic velocity
q-PCR	Quantitative real-time polymerase chain reaction
RNA-seq	RNA sequencing
ROS	Reactive oxygen species
SA	Spiral arteries
SBP	Systolic blood pressure
SEM	Standard error of the mean
TAC	Total antioxidant capacity
UA	Umbilical artery
