# Supplementary material for: SUMOylation Inhibitor TAK-981 Alleviates Hallmark Features of Preeclampsia Related to High Glucocorticoid Exposure by Inhibiting Placental Oxidative Stress in Rats
Source: Antioxidants (Basel). 2026 Apr 11;15(4):478. doi: 10.3390/antiox15040478 (PMC13113754; doi:10.3390/antiox15040478)
Supplement: Supplementary file 1 [file antioxidants-15-00478-s001.zip › antioxidants-4176439-supplementary.pdf]

Online supplement tables and figures

Table S1. Clinical characteristics of the pregnant woman enrolled in this study

	CON (n=18)	PE (n=24)	P value
Maternal age (years)	33.46±3.926	33.37±4.051	0.5658
BMI (kg/m^2)	25.32±2.953	29.56±4.297	0.0013
Gestational age (wk)	39.14±0.596	37.21±1.401	＜0.0001
Systolic blood pressure (mmHg)	109.6±14.03	157.3±15.05	＜0.0001
Proteinuria (g/24h)	NA	3.565±2.694	NA
Fetal weight (g)	3369±259.4	2662±599.3	＜0.0001

Statistical analysis was performed by two-tailed Student’ s t test.

Table S2. Antibodies for WB, IF and IP.

Antibody	Manufactory	Catalog Number
α-SMA	Servicebio	GB13044
β-actin	Proteintech	20536-1-AP
NDUFS1	Abclonal	A21192
Pan-CK	Santa Cruz	SC-81714
SDHB	Proteintech	10620-1-AP
UQCRC2	Proteintech	14742-1-AP
SUMO1	Abclonal	A2130
SUMO2/3	Abclonal	A22734

Table S3. Primers sequence for q-PCR.

Primers	Forward Primer (5' -> 3')	Reverse Primer (5' -> 3')
β -actin (Rat)	CGTAAAGACCTCTATGCCAACA	TAGGAGCCAGGGCAGTAATC
Senp1 (Rat)	AATGCAGCGTGCCTAGAAGT	CACACTGCAACCTGCTGAAC
Senp3 (Rat)	ACCACAGTGCCAACTAGACG	TGAGAGGAGACCCGAGTCAG
Senp7 (Rat)	ATGCCCCAAAGTCCCTAAGC	GCAAACCTGACAAGCACTGG
Sumo1 (Rat)	TCTGACCAGGAGGCAAAACC	ATGCACAGTCCTGGAAAGGG
Sumo4 (Rat)	CCGACGAGAAACCCAAGGAA	ACCAGAACTGTCCCCAGACT

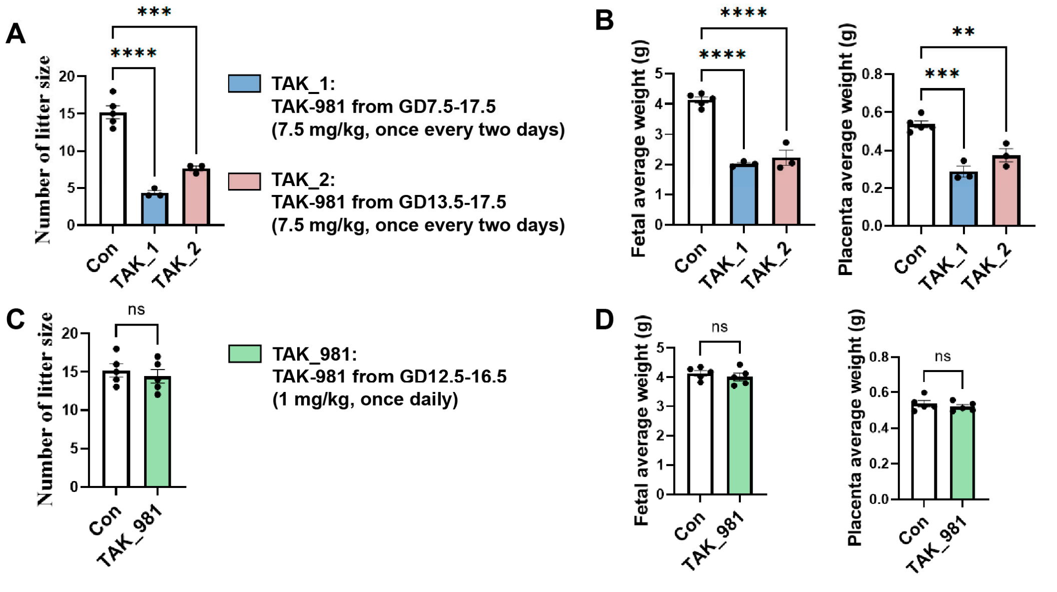

Figure S1. The Effects of different doses of the SUMO Inhibitor TAK-981 on litter size, fetal weight and placental weight. (A) little size measured on GD 20.5. Error bar, SEM, n=5 in Con group, n=3 in TAK_1 and TAK_2 groups. **(B)** fetal and placental average weight from dams measured on GD 20.5. It represents mean fetal and placental weight from each dam. Error bar, SEM, n=5 in Con group, n=3 in TAK_1 and TAK_2 groups. **(C)** little size measured on GD 20.5. Error bar, SEM, n=5 in each group. **(D)** fetal and placental average weight from dams measured on GD 20.5. It represents mean fetal and placental weight from each dam. Error bar, SEM, n=5 in each group. ** $p < 0.01$, *** $p < 0.001$, **** $p < 0.0001$, ns: not significant.

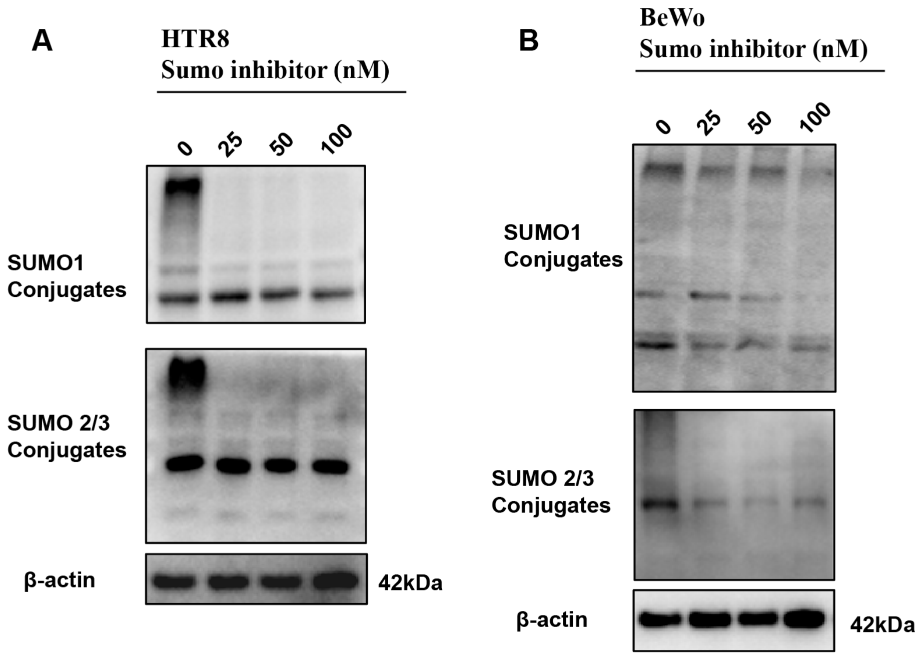

Figure S2. Sumo inhibitor effectively suppressed cellular SUMOylation. HTR8 cells were treated with Sumo inhibitor (25nM, 50nM, 100nM) for 24h. BeWo cells were treated with Sumo inhibitor (25nM, 50nM, 100nM) for 48h. The cells were then harvested for WB. (**A, B**) Protein levels of SUMO1 and SUMO2/3 conjugates in HTR8 cells (**A**) and BeWo cells (**B**).

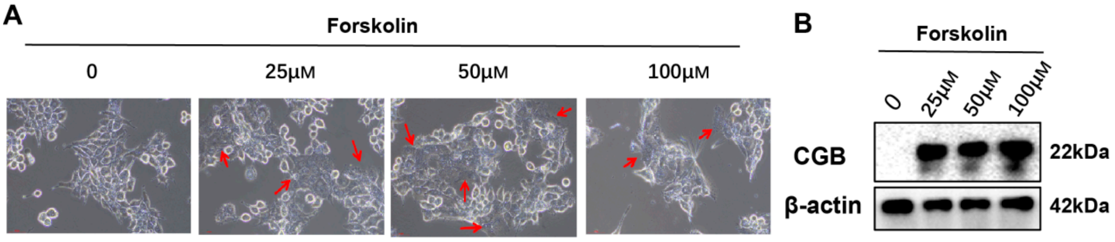

Figure S3. Forskolin induced syncytialization in BeWo cells. BeWo cells were treated with forskolin (25 μ M, 50 μ M, 100 μ M) for 48h, meanwhile. The cells were then used for the syncytialization analysis. In some cases, cells were harvested for WB analysis. (**A**) representative syncytialization images of BeWo cells. (**B**) Protein levels of CGB (quantifiable syncytization) in BeWo cells.
